# Phylostat: a web-based tool to analyze paralogous clade divergence in phylogenetic trees

**DOI:** 10.3906/biy-2105-18

**Published:** 2021-12-14

**Authors:** Elif ÖZÇELİK, Nurdan KURU, Ogün ADEBALİ

**Affiliations:** 1 Molecular Biology, Genetic and Bioengineering, Faculty of Engineering and Natural Sciences, Sabancı University, İstanbul Turkey

**Keywords:** Phylogenetics, paralog, ortholog, divergence, statistics, phylogenetic tree, visualization, vector graphics, web server

## Abstract

Phylogenetic trees are useful tools to infer evolutionary relationships between genetic entities. Phylogenetics enables not only evolution-based gene clustering but also the assignment of gene duplication and deletion events to the nodes when coupled with statistical approaches such as bootstrapping. However, extensive gene duplication and deletion events bring along a challenge in interpreting phylogenetic trees and require manual inference. In particular, there has been no robust method of determining whether one of the paralog clades systematically shows higher divergence following the gene duplication event as a sign of functional divergence. Here, we provide Phylostat, a graphical user interface that enables clade divergence analysis, visually and statistically. Phylostat is a web-based tool built on phylo.io to allow comparative clade divergence analysis, which is available at https://phylostat.adebalilab.org under an MIT open-source licence.

## 1. Introduction

Gene duplication is the primary mechanism in evolution to innovate new proteins (Long et al., 2003). In his famous book *Evolution by Gene Duplication,* Ohno proposed that after gene duplication, one of the two copies accumulate mutations, which may lead to the invention of a new gene (Ohno, 1970). The homologous sequences that are products of a gene duplication and speciation are known as paralogs and orthologs, respectively. Gene duplication results in one of the following scenarios: (i) If both paralogs are selected and do exist today, it is often that one of the duplicates conserved the parental function and the other copy diverged and gained a partial or complete new function (neofunctionalization); (ii) One of the copies accumulates mutations and become a functionless pseudogene (non-functionalization); (iii) Both duplicates complement each other’s function and, therefore, are both selected (sub-functionalization) (He and Zhang, 2005). In such a case, a parental version of the duplicates does not exist. Neo-functionalization and sub-functionalization give rise to an innovation. Therefore, it is improbable for both paralogs to conserve the ancestral function. Consequently, two genes that once shared the same sequence and protein product are likely to have functionally diverged from each other due to the nature of evolution where redundancy is disfavored (Nowak et al., 1997).

Though it is straightforward to establish evolutionary histories for the gene families with one-to-one relationships, it is not rare to observe extensive gene duplications for modular gene families. When the number of duplications is high, the functional relationships between homologs become difficult to establish. Despite being widely adopted terms, orthologs and paralogs might remain insufficient in distinguishing functionally diverged homologs. Orthology and paralogy do not necessarily indicate functional associations; they are yet frequently used as indicators of functional equivalence and divergence, respectively. However, further specifications are necessary to uncover the entire evolutionary relationships between homologous genes to gain more insight with respect to their function. Especially between co-orthologs, which are the genes orthologous to another gene or genes created as a result of gene duplication after speciation, it might be possible to further dissect the phylogenetic trees and identify the common ancestral function between paralogous clades. Orthologs that are not affected by the accelerated rate of mutation accumulation are termed *primary orthologs *(Lafond et al., 2018). Equivalently, orthologs conserving the last common ancestral function were termed *isorthologs *(Swenson and El-Mabrouk, 2012).

Essential genes are vital for organismal survival and conserved throughout millions of years of evolution. Loss-of-function of those critical genes results in either severe disease or mortality (Bartha et al., 2018). Gene duplication and loss events complicate evolutionary history of a gene. Lineage-specific events result in incomprehensible functional relationships between co-orthologs (Gabaldon and Koonin, 2013). However, for genes that are commonly essential for the organisms under investigation, following gene duplication, one of the duplicates likely maintains the ancestral function, whereas the other version is free to diverge. Purifying selection pressure often acts on only one of the duplicates because its function is necessary and sufficient to maintain the fitness. Therefore, it is tempting to parse lineage specific duplications and reveal the common function-wise ancestral version of the duplicates, which potentially conserved the parental function, with the aim of possibly obtaining functionally equivalent orthologs across lineages each with independent gene duplications.

Phylogenetic trees are visualized to infer evolutionary relationships between homologous entities, which can be genes, proteins, and species. There is a number of tools for phylogenetic tree visualization. FigTree (http://tree.bio.ed.ac.uk/software/figtree/) stands out as one of the popular tools that is locally installed on any operating system. Dendroscope is another tree visualization tool that is popular among biologists (Huson and Scornavacca, 2012). Along with command-line tree analysis and manipulation features, ETE tools (Huerta-Cepas et al., 2016) also provide a tree visualization platform. This tool is highly useful especially for aligning the corresponding features, such as sequences, with the leaves in the tree. Another visualization tool is embedded in a comprehensive molecular evolution software MEGA (Kumar et al., 2018). Phylogenetic visualization feature of MEGA complements its powerful evolutionary analyses. Finally, there are installation-free browser-based applications. These tools are mainly phylogeny.io (Jovanovic and Mikheyev, 2019), phylo.io (Robinson et al., 2016), icytree.org (Vaughan 2017), iTOL (Letunic and Bork, 2007). Although these tools provide extensive visualization capabilities, they do not provide further graphical user interface enabling further inference capabilities on the trees. 

With the current phylogenetic techniques, it is feasible to infer about the evolutionary process of a gene by understanding molecular evolution. Here, we developed Phylostat that allows pairwise comparison of selected clades and applies phylogenetic tests in the context of protein sequence comparisons to determine whether one of the paralogous clades is differentially closer to the common ancestor.

## 2. Methods

We have built Phylostat on an existing software, phylo.io (Robinson et al., 2016). We added functions in javascript to allow multiple node selection, coloring, and tests. The framework of Phylostat consists of two comparative tests between two selected clades that are diverged from each other through gene duplication. The aim of the statistical test is to estimate whether one of the duplicates potentially retained the ancestral function or not. If a duplicate preserves the original function, Phylostat helps the user to detect which of the duplicate is the one closer to the ancestor. 

### 2.1. Internal divergence in a clade

After a gene duplication occurs in an extinct organism, one of the duplicates can diverge throughout generations and gain a new function. The neofunctionalized paralog might have gained an essential function, and its absence would not be favored for survival. In such a case, although the neofunctionalized version of the two paralogs is diverged from the “original” one, its divergence can be limited during speciation. Such phenomena show that although the paralogs are differentially diverged from the common ancestor, they did not diverge differentially within the clade during speciation. To calculate the divergence within the clade, Phylostat takes the individual branch lengths and compare the divergence rates within the clade. If in-clade divergence between two paralogous clades differs from each other, one can hypothesize the differential variation between two paralogs. This criterion differs from the “pairwise-distance approach”, which is based on comparing leaf-to-leaf distances within clades that we previously presented (Adebali et al., 2016). Although both methods can be used to understand the divergence during speciation, in case of the existence of outliers among the branch lengths, our current approach counts them only once. Phylostat takes all branch lengths in clades and stores them as a set. It performs t-test to assess whether two sets of internal distances are different from each other. Although Student’s t-test provides a powerful statistic in case the differences are normally distributed and the variance of two groups is equal, these assumptions are not always met. Especially, it is reasonable to expect various type of tree topologies since the tool works on user-defined trees. By taking this diversity of the trees and analysis into account, Welch’s t-test and Mann–Whitney U test are implemented to cover unequal variance and non-normality of the differences, respectively. The related test statistic and *p* value is provided on the webpage. If species names are defined in the leaves, user can input a regular expression pattern to identify the unique id (or name) of the species. When this option is used, the comparison is made by using the common species between the two clades, and the branch lengths are updated with respect to the pruned clades, which only includes the common species of the two clades in comparison. 

### 2.2. Species sets

If a gene is essential for survival, its loss would result in a significant cost in fitness. Therefore, species lacking an essential gene cannot survive. After duplication of an essential gene which cannot be deleted with no fitness loss, at least one of the versions of the duplicates must conserve the original function. After most of the gene duplication events, a duplicate is pseudofunctionalized and does not express any protein. For some cases though, the second non-essential duplicate gains a new function, which may or may not provide additional evolutionary benefit to the organism. In such a case, the second gene can be dispensable for some species. Because of a tolerable absence of the duplicate, some species lose it with no fitness cost. Therefore, to test which one of the duplicates is essential and which is not, the species contents between two clades should be considered. If a phylogenetic tree contains the species information, such as taxonomic id, in the name of the leaves, Phylostat can perform species content analysis. Users specify a Regex syntax to provide where in the leaf name the organism information is stored. Phylostat plots a Venn diagram showing the species content of each clade. If a clade is superset of the other one, this suggests that the superset clade is likely be more essential than the other clade. The differential genome content between two clades suggests complementarity between two genes; either one of the duplicates may be sufficient for the species. 

When unique identifier of the species is defined by the user using regular expression patterns, Phylostat applies the branch divergence tests on the common species only. When there are multiple genes/proteins belonging the same species in one clade, Phylostat chooses a representative, which would be the least diverged leaf based on their distance to the root. The rationale of this feature is to detect the least diverged clade and more diverged version in a single specie lineage might introduce false or undesired evolutionary signals due to either less or no natural selection pressure (neo/sub/non-functionalization) or sequencing errors. This feature is useful for paralogous sequences in the clade as well as isoforms that are usually present in the trees generated with sequences obtained from an online Blast search.

The boxplot was plotted with plotly.js which is licensed under MIT license. The Venn diagram was drawn with Highcharts, which can be used for non-commercial purposes freely. All plots can be downloaded at high resolution in SVG format. Images can also be downloaded in noneditable png format for a quick representation. 

## 3. Results

### 3.1. Gene duplication analysis – test cases

In this section, we exemplified different test cases in order to illustrate the usage of Phylostat.

**Figure 1:**
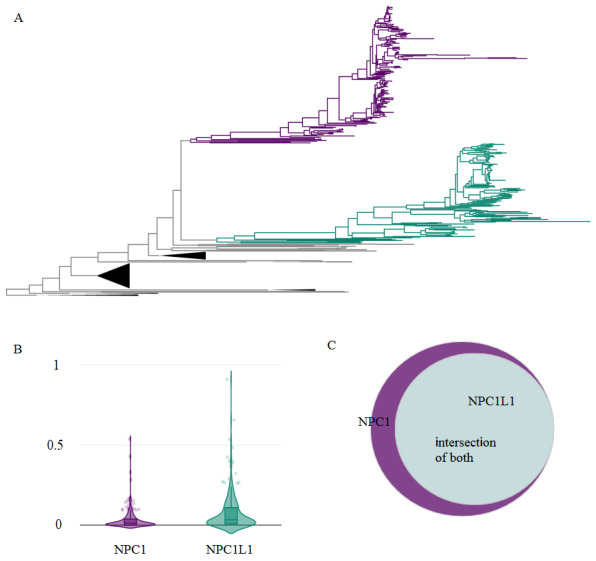
Clade divergence analysis between NPC1 and NPC1L1. (A) Phylogenetic tree of NPC1 (purple) and NPC1L1 (green). Regex expression, “taxid.[0-9]+” is used to identify unique species. (B) Internal branch length comparison of the clades (see section 2.2). NPC1L1 clade has higher internal distance values with t value –5.90704 and p value 6.10049e-9. Thus, we can only provide that p value is smaller than this threshold. (C) Venn diagram shows that NPC1 species set is the superset of NPC1L1 with 40 unique species. There are 210 leaves in the NPC1 clade and 152 leaves in the NPC1L1 clade.

In the first test case, we examined the protein tree of NPC1 (Figure 1), which is a gene that is associated with Niemann–Pick disease Type C (Vanier, 2010), a rare Mendelian disease. Previously we have shown that humans as well as most other jaw vertebrates have a paralog of this gene called NPC1L1 (Adebali et al., 2016). Unlike NPC1, NPC1L1 has no association with any Mendelian disease. After uploading the tree, we selected two clades with respect to the two human paralogues they involve, NPC1 and NPC1L1, to evaluate which of the paralogs could be considered as the function-wise ancestral version. The test shows that NPC1L1 is internally more diverged than NPC1 (Figure 1B). Moreover, species sets show that NPC1 clade is a superset of NPC1L1 (Figure 1C) since NPC1 clade has 40 unique species in addition to the 119 common species in two clades. With the information that Phylostat provides, it could be inferred that NPC1 is closer to the function-wise ancestral version of these clades from a phylogenetic perspective. The p value of the comparative test is significant, as previously reported (Adebali et al., 2016).

**Figure 2:**
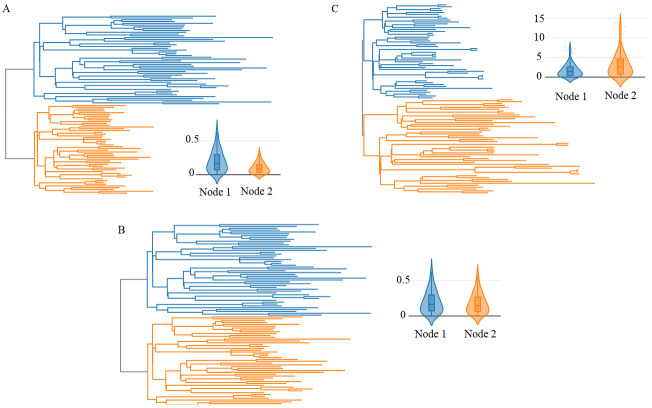
Clade and internal divergence analysis for simulations. The statistical analysis results can be found in Table. (A) The first scenario covers the case where the first clade is more diverged. (B) The second scenario represents two equally diverged clades. (C) In the last example, two clades show different trends in terms of divergence rates, like the first one where the second clade is more diverge

For further tests on the performance of Phylostat, some MSAs were simulated using ALF (Dalquen et al., 2012), which is a tool designed for simulating sequence evolution by considering various evolutionary forces that act on genomes such as indels, gene duplication, gene loss etc. We construct three simulation experiments with ALF under different mutation, birth, and death rates. To obtain realistic results, gamma distributed rate variation among sites is used, and LG is preferred as amino acids substitution matrix. ALF has more than 60 parameters to adjust the tree topology, sequence of the root genome, insertion and deletion rates, duplication rate, etc. We employed the related parameters depending on our aim of obtaining duplication node or simulating without any duplication, but the remaining parameters are taken as 0 (such as genome rearrangement, ratio of translocation and rate of fission after duplication) or left as default (such as insertion and deletion rates, gene loss). The resulting trees, scores, and test statistic values are reported in Figure 2 and Table, respectively. Each of the trees in Figure 2 is a result of three individual simulations. The first part of the simulation aims to obtain a gene duplication process. With the help of increasing the duplication rate to 0.05 from the default value of 0.0005, we obtain a small tree with a pair of paralog genes. In these experiments, the number of proteins that the first organism have is taken as 1, mutation rate is taken as greater than 1000, birth and death rates are left as default, which are 0.01, 0.001, respectively. Although ALF provides alignment, gene and species trees, the gene tree is ultrametric, which means all leaves are equidistant from the root, which is not a realistic assumption. By taking the MSA constructed by ALF, we reproduce a maximum-likelihood tree by using RAXML-NG (Kozlov et al., 2019). The second and third scenarios are based on using the sequence of paralog genes as “root genome” and modelling the evolution under given ancestral genome. In these simulations, to produce no duplication node and paralog-free clades, the duplication rate is taken as 0. Additionally, the number of resulting species is increased to 30 to obtain meaningful results for the divergence test. ALF generates random names for species produced at the end of simulations. Since it is not possible to detect the common species between two clades, we use the same tree topology for the second and third simulations. Although the general topology is the same, the branch lengths are determined by the mutation rate. By enforcing the tree topology, we match the species from Clade 1 to Clade 2. Figure 2 includes some representative results over various scenarios related to the criteria. The results of superset criterion are not reported since two clades have the same number of species and completely overlap for all three examples. 

**Table:** The p values of test statistics for simulated datasets. In the first simulation, the first clade is internally more diverged than the second one, and in the third simulation it’s the other way around. However, as can be seen from the results, the difference in simulation 1 is more significant than simulation 3, but simulation 3 rejects the null hypothesis. For the second simulation, the overall divergence of internal distances is not significantly different for two clades.

DATA	Internal divergence		
	t-test	Welch’s t-test	Mann–Whitney U test
Simulation 1	8.35e-8	8.35e-8	0.0000052
Simulation 2	0.35	0.35	0.40
Simulation 3	0.000071	0.000071	0.00067

## 4. Discussion

Visual interpretation of phylogenetic trees does not yield the entire evolutionary information regarding the genes, proteins, or species. In order to lay out the inferred feature in a statistical context, we presented a web-based tool allowing manual and automated statistical inference. The manual part of the software is the choice of the clades of interest by the user. After users select the clades, the tool automatically computes the statistical features and results of the first criteria. The null hypothesis is that two clades do not diverge differentially from each other. Phylostat outputs statistical evaluation for rejecting the null hypothesis.

Each gene has a unique evolutionary history. Phylogenetic analyses are almost always coupled to manual inference. The automated approaches limit our ability to infer protein-specific features. However, we lack human power to carefully analyze the evolutionary history of thousands of protein-coding genes. Therefore, it is important to develop partly automated approaches that take into account gene-family specific features. If these features are defined well, it would be possible to develop automated phylogenetic inference tools. With such tools in hand, the protein families will be better categorized, and researchers will be enabled to perform fine-tuned experiments. With the aim of constructing high-resolution phylogenetic trees, evolutionary events should be precisely defined. The methods proposed here will be utilized to test the existence of a function-wise parental copy of duplicates and, if it exists, to determine which of the paralogs retained the ancestral function. After thorough analyses of gene families that are representatives of the genome in terms of evolutionary history, these methods can be implemented in a robust pipeline to annotate evolutionary relationships of homologous sequences. As of now, Phylostat substantially contributes to the clade divergence visualization and statistics for a single tree and single node at once. Users need to be aware of the gene duplication node and select it for further analysis. It is not possible to interpret the automated clade-to-clade comparison for all gene duplication nodes. In the future, we aim to enhance the tool by adding new features. The source code of Phylostat is available (https://github.com/CompGenomeLab/phylostat), and its repository is open for contributions. A well-documented repository and active support unit through GitHub issues are available to enhance collaborations.

### 4.1. Suggestions for phylogenetic tree reconstruction

Several approaches to generate phylogenetic trees are as follows: neighbor joining, maximum parsimony, maximum likelihood, and Bayesian inference (Horiike, 2016). It has been shown that the most accurate trees are generated through maximum likelihood and Bayesian inference methods (Ogden and Rosenberg, 2006). Although these two methods are computationally expensive for a precise analysis such as the ones we illustrated in this study, we recommend using these accurate methods. We also recommend users who work on protein sequences to retrieve one isoform for each gene per taxon. Including more than one isoform in a phylogenetic tree is irrelevant and might result in deviating outcomes particularly when testing the internal divergence with no species name specified. As the best practice, we recommend including a unique ID for each genome that gene or protein belongs to. If species are not defined, tests are performed using all the leaves, and inclusion of paralogs might result in redundancy and meaningless comparisons. Taxonomic ID (NCBI) (Schoch et al., 2020) has been a standard for these types of comparative genomics studies. Taxonomic IDs can also be used to label the nodes with intermediate taxonomic levels, which might give additional insight into where gene duplication/deletion occurred. Although bootstrapping requires additional layers of computation especially for computationally expensive methods such as maximum likelihood and Bayesian inference, they will help to assign the duplication nodes confidently. 

## References

[ref1] Adebali O Reznik AO Ory DS Zhulin IB 2016 Establishing the precise evolutionary history of a gene improves prediction of disease-causing missense mutations Genetics in Medicine 18 1029 1036 26890452 10.1038/gim.2015.208 PMC4990510

[ref2] Bartha I di Iulio J Venter JC Telenti A 2018 Human gene essentiality Nature Reviews Genetics 19 51 62 10.1038/nrg.2017.75 29082913

[ref3] Dalquen DA Anisimova M Gonnet GH Dessimoz C 2012 ALF—a simulation framework for genome evolution Molecular Biology and Evolution 29 1115 1123 22160766 10.1093/molbev/msr268 PMC3341827

[ref4] Derrick B Russ B Toher D White P 2017 Test statistics for the comparison of means for two samples that include both paired and independent observations Journal of Modern Applied Statistical Methods 16 9 9

[ref5] Gabaldon T Koonin EV 2013 Functional and evolutionary implications of gene orthology Nature Reviews Genetics 14 360 366 10.1038/nrg3456 PMC5877793 23552219

[ref6] He X Zhang J 2005 Rapid subfunctionalization accompanied by prolonged and substantial neofunctionalization in duplicate gene evolution Genetics 169 1157 1164 15654095 10.1534/genetics.104.037051 PMC1449125

[ref7] Horiike T 2016 An introduction to molecular phylogenetic analysis Robotics and Autonomous Systems 4 36 45

[ref8] Huerta-Cepas J Serra F Bork P 2016 ETE 3: Reconstruction, Analysis, and Visualization of Phylogenomic Data Molecular Biology and Evolution 33 1635 1638 26921390 10.1093/molbev/msw046 PMC4868116

[ref9] Huson DH Scornavacca C 2012 Dendroscope 3: An Interactive Tool for Rooted Phylogenetic Trees and Networks Systematic Biology 61 1061 1067 22780991 10.1093/sysbio/sys062

[ref10] Jovanovic N Mikheyev AS 2019 Interactive web-based visualization and sharing of phylogenetic trees using phylogeny IO. Nucleic Acids Research 47 W266 W269 31114886 10.1093/nar/gkz356 PMC6602505

[ref11] Kozlov AM Darriba D Flouri T Morel B Stamatakis A 2019 RAxML-NG: a fast, scalable and user-friendly tool for maximum likelihood phylogenetic inference Bioinformatics 35 4453 4455 31070718 10.1093/bioinformatics/btz305 PMC6821337

[ref12] Kumar S Stecher G Li M Knyaz C Tamura K 2018 MEGA X: Molecular Evolutionary Genetics Analysis across Computing Platforms Molecular Biology and Evolution 35 1547 1549 29722887 10.1093/molbev/msy096 PMC5967553

[ref13] Lafond M Meghdari Miardan M Sankoff D 2018 Accurate prediction of orthologs in the presence of divergence after duplication Bioinformatics 34 i366 i375 29950018 10.1093/bioinformatics/bty242 PMC6022570

[ref14] Letunic I Bork P 2007 Interactive Tree Of Life (iTOL): an online tool for phylogenetic tree display and annotation Bioinformatics 23 127 128 17050570 10.1093/bioinformatics/btl529

[ref15] Long M Betran E Thornton K Wang W 2003 The origin of new genes: glimpses from the young and old Nature Reviews Genetics 4 865 875 10.1038/nrg1204 14634634

[ref16] Nowak MA Boerlijst MC Cooke J Smith JM 1997 Evolution of genetic redundancy Nature 388 167 171 9217155 10.1038/40618

[ref17] Ogden TH Rosenberg MS 2006 Multiple sequence alignment accuracy and phylogenetic inference Systematic Biology 55 314 328 16611602 10.1080/10635150500541730

[ref18] Ohno S 1970 Evolution by gene duplication

[ref19] Robinson O Dylus D Dessimoz C 2016 Phylo.io: Interactive Viewing and Comparison of Large Phylogenetic Trees on the Web. Molecular Biology and Evolution 33 2163 2166 27189561 10.1093/molbev/msw080 PMC4948708

[ref20] Schoch CL Ciufo S Domrachev M Hotton CL Kannan S 2020 NCBI Taxonomy: a comprehensive update on curation, resources and tools Database 1 21 32761142 10.1093/database/baaa062 PMC7408187

[ref21] Swenson KM El-Mabrouk N 2012 Gene trees and species trees: irreconcilable differences BMC Bioinformatics 13 Suppl 19 S15 S15 10.1186/1471-2105-13-S19-S15 PMC3526438 23281654

[ref22] Vanier MT 2010 Niemann-Pick disease type C Orphanet Journal of Rare Diseases 5 16 16 20525256 10.1186/1750-1172-5-16 PMC2902432

[ref23] Vaughan TG 2017 IcyTree: rapid browser-based visualization for phylogenetic trees and networks Bioinformatics 33 2392 2394 28407035 10.1093/bioinformatics/btx155 PMC5860111

